# Collections of Morbid Anatomy

**Published:** 1814-04

**Authors:** J. C. Warren


*For the Medical and Physical Journal.*

*Collections of Morbid Anatomy; by* J. C. Warren, M.D.

*INFLAMMATION of the Pericardium.*—In the month of February, 1813, a number of individuals in Boston were attacked with violent pains in the side, or breast, or upper part of the abdomen. These pains were, in many instances, like those from tearing or rending asunder the parts affected. They were rarely accompanied with elevation of the pulse, and in the intervals of pain, the pulse was commonly slow and always feeble. The affections of the abdomen were relieved in two or three days, by purgatives and blistering; those of the thorax, when slight, by bleeding and blistering; but a number of the latter assumed a most violent and terrible character, and proved fatal to the patient, in three or four days. At the period when these disorders were appearing, the two followiag cases occurred.

The subject of the first was a distinguished gentleman of the clergy, who was possessed of a most placid and amiable disposition, which no event could disturb. His health had been good, for three or four years, excepting a degree of indigestion, not sufficient to induce him to mention it frequently. At the period of his attack, he had been dining in company, and had retired to another room, when he was suddenly seized with a most violent pain in the breast, that threatened immediately to deprive him of life. This pain extended from the breast to the arms. He could express the sensation only by saying, that the limbs seemed tearing and twisting from the body. Sweat poured in streams from every part, while the skin was universally of a death-like coldness. He had an emetic, which produced very copious evacuations, and was followed by a mitigation of the pain. The next day the patient seemed better, and was able to converse; but it was evident that his constitution had received a severe stroke. He said that there was some extraordinary difficulty about his heart, and requested that, after his death, an examination of that organ might be made. His tongue was coated of a yellowish white color. His pulse extremely irregular. No heat of the skin, although he sometimes complained of a sensation of heat. This day he had a cathartic, and remained free from any severe pain till toward evening, when he had another attack. On the third day of the disease, he had repeated paroxysms of the most excruciating pain, which brought on a convulsive action of the whole system and a fainting, that was followed by relief from the distress. His pulse was now quite imperceptible.

His

His muscular powers were still so considerable, that except to the eye of the physician, the fatal tendency of his complaint was not very perceptible to himself or others. It was announced to him that he must die.. He expressed a little surprise at this intelligence, but exhibited no emotion of alarm, and calling his family about him, he gave directions respecting his affairs and his funeral, and having taken leave of them, tranquilly resigned himself to his fate. After this time, he lingered still thirty hours longer, without any remarkable symptoms, for the fatal blow was already struck. He remained, almost to the last, in a semi-recumbent posture, and possessed the faculties of mind to the last moment.

It is impossible to avoid comparing the circumstances of this case with those of the celebrated Mirabeau, described in our last number. They both perished of the same disease. But the manner in which the atheist expired excites horror, while the death of this truly christian clergyman inspires us with admiration.

The body was examined, on the day after death, agreeably to the request of the deceased. No remarkable external appearance presented itself. In the cavity of the chest, the lungs exhibited a natural and healthy appearance; and their vessels were very moderately charged with blood. The mucous membrane of the bronchiæ had a slight appearance of inflammation. The pleura was not inflamed, but exhibited a net-work of vessels in various parts, which probably would have gone on to decided inflammation. The cavities of the pleura contained a little serous fluid. The loose pericardium was covered with a uniform but delicate blush, deepest near the diaphragm. The pericardium, which closely invests the heart, exhibited marks of violent inflammation. It was generally of a red color, but this color became of a livid hue on the surface next the diaphragm, like the color of parts tending to gangrene. This membrane was thickened, and in many parts covered with coagulated lymph. A small quantity of serum, mixed with semi-purulent lymph, was contained in its cavity. The substance of the heart was swelled, and remarkably tender. It was covered with fat. None of its cavities were enlarged. They were all filled with blood, which was principally fluid. The semilunar valves of the aorta had the hardness of a fibro-cartilage, and in some places were in a state near ossification. The inner coat of the aorta was brittle, and contained a number of hard specks. The organs in the cavity of the abdomen were in a healthy state, and exhibited no very remarkable appearance. The omentum was loaded with fat. The veins of the stomach and intestines were very conspicuous, and appeared

 flaccid, flaccid, as if they had been over-distended. The mucous coat of the stomach exhibited a slight redness. Its cavity contained fluid, and a considerable quantity of solid matter, which seemed to be half-digested animal food. In the midst of this was a large living round worm.*

Inflammation of the pericardium and inflammation of the heart should, no doubt, be considered as one disease. They are both inflammations of the serous membrane. It seems probable, however, that an inflammation of the close investment of the heart will be marked with a more decided character, than that of the external pericardium. Most of the symptoms of this disease are exhibited in the case related, with the most formidable and strongly marked aspect. The distress about the diseased organ, the peculiar and agonizing pain in the arms, the irregular and sinking pulse, and the disposition to fainting, are not to be found combined in any other acute disease. One important symptom was absent, the febrile affection which accompanies inflammatory diseases. This peculiarity may be explained; for it is this absence of febrile appearance, together with the violence of invasion, the suddenness of termination, and the

---

* An interesting case of this kind occurred in the practice of Mr. Want. It commenced with the most agonizing spasmodic affection of the chest and pit of the stomach, which required extraordinary doses of opium to relieve. It was *unfortunately* not at the time suspected to be inflammatory. Rheumatic pains of the limbs supervened; and after this the chest became again affected, and seemingly by metastasis, as the pains left the limbs. At this time, the difficulty of breathing and tightness across the præcordia was peculiarly severe; the heart began to palpitate incessantly; and the pains about the stomach returned with great violence. Blood drawn from the arm was highly inflamed: the bleeding was repeated daily, and sometimes twice in the day, till the patient died. This operation was always followed by relief more or less lasting. During the course of the disease the symptoms continued nearly the same, excepting only the temporary diminution in violence after each bleeding. A few days before death, constant vomiting and faintings were superadded to the general distress.

On dissection, a quantity of fluid was found in the cavities of the pleura; but the principal morbid appearances were discovered in the heart. It was greatly inflamed, with spots of coagulable lymph on its surface. The cavities of the right side were filled with polypous concretions of a transparent yellowish color, and of a consistence possessing nearly the firmness of cartilage. Adhesions to the pericardium had taken place in some parts. Its cavities contained about two ounces of fluid. The inner surface of the stomach was also slightly inflamed.

livid livid aspect of the inflamed parts, which induce us to refer this disease to the epidemic, that prevailed in various parts of the country, and just showed itself in the metropolis. We cannot attribute any important consequences to the diseased state of the valves of the aorta.

*Inflammation of the Pleura, with ulceration.*—This case is not to be considered as a common inflammation of the pleura, although we have assigned it that name. The symptoms, as well as the morbid appearances, demonstrate a malignity of disease, quite peculiar, and which would perhaps entitle it to a very different name from that assumed. The invasion of disease was as sudden and violent as in the former case. The subject was a gentleman, in the prime of life, possessed of a most powerful muscular apparatus, an ample frame of body, and an iron constitution. About two years before his death, he felt some dyspeptic symptoms, which, among other unpleasant sensations, caused a vertigo, extremely alarming to the patient. He became hypochondriac, and conceiving that exposure to the air might be pernicious, he confined himself almost wholly to his house, and totally relinquished his accustomed exercise; while, at the same time, his appetite being unimpaired, he satisfied it as freely, as though he had no complaint. This course of life undoubtedly increased the symptoms of indigestion, which only confirmed him in the mode of living he had adopted. Yet it is remarkable, that while he laboured under these false and melancholy impressions respecting himself, his wit, which was natural and ready, scarcely ever languished. After remaining in this state nearly two years, contrary to his usual custom he quitted his home, in the evening, to visit his friends in the adjoining house. This slight exposure was the cause of his disease and death; so much was his constitution enervated by long confinement. In the night, he was attacked with an agonizing pain in the left side, which frequently remitted, or, as he thought, he must have expired from its severity. Various applications were made on that night and the following day, the principal of which were evacuants, and liquid cantharides to the seat of pain. He at last got relieved, and was well enough on the third day to make considerable exertions; but on the evening of that day the pain returned, with dreadful violence. He had also a cough, considerable fever, and an incessant inclination to pass urine, from the absorption of the cantharides. To alleviate his sufferings, very frequent doses of opiate medicines were given that night and the following day, but so long without effect that he had taken a considerable quantity before obtaining relief. At last he slept, and remained quiet for for the night. In the morning following, when it was expected he would be found better, he did not awake. Efforts were made to rouse him, but in vain; the intellectual powers were irrecoverably gone. When he was raised in bed, an enormous tumour was discovered on the side of the neck, hard as a stone, and filling the neck almost from the ear to the clavicle. In the night he expired.

On examination of the body, two days after death, the first thing observed, to the astonishment of all, was the disappearance of the great tumour on the neck. No vestige of it remained in the part where it had been seen, but below the inferior jaw, and extending parallel to it, there was a slight and scarcely observable thickening of the cellular membrane. On cutting into this cellular membrane, it appeared firm and somewhat *crisp*. The parotid gland of the left side was immediately examined, but its appearance was perfectly healthy. Next, the submaxillary gland, which was a little enlarged, harder than usual, and internally of a dark colour. The other appearances, on the surface of the body, were a yellowness of the skin, and a thick deposition of fat in every part of the cellular membrane. The thorax being struck, resounded every where, except in the part which was the seat of pain, and here the sound was dull. The muscles cut into, were generally of a darker colour than common, but not so conspicuously livid as in cases of the disease called petechial fever. The veins of the brain were very full of fluid blood. The arachnoid coat contained a few spots of coagulated lymph, and between this and the pia mater was a little serous fluid. The substance of the bra n was quite soft, and its medullary portion was stained, when cut, with the fluid blood which oozed from its small vessels. The lungs were no sooner exposed than they were seen to be the seat of a very peculiar appearance. The right lung had six or seven spots, very similar to those produced on the skin by the operation of a moderate caustic. These spots were between an inch and the eighth of an inch in diameter. In all, the edges were whitish and elevated; in some, they were separated from the surrounding pleura. The substance of the lungs under these spots was very hard and red to some depth. The left lung adhered to the pleura of the ribs for an extent of three inches. When this adhesion was separated, a very thick layer of coagulated lymph was exposed, between the pleura of the lungs and that of the ribs; and on the outer surface of this lymph, that is, between it and the pleura of the ribs, was a shallow cavity, containing a semi-purulent liquid. Smaller adhesions and cavities were seen in other parts of the lung. At all these adhesions, the

 pleura pleura was not merely dark or livid, but absolutely black, and approaching to gangrene. In the left cavity of the pleura, a quantity of liquid blood was effused. That it was not discharged from the lungs, when the adhesion to the ribs was separated, I cannot positively determine, but believe that it was effused before death. The pleura, on the surface of the left lung, was wrinkled, as if it had been exposed to fire, probably from a deficiency of its serous exhalation. The substance of the lungs was hard, swelled and gorged with blood. The heart was fat. Its cavities contained a moderate quantity of liquid blood. In other respects, this organ and its appendages were perfectly healthy. The mucous coat of the stomach was uniformly reddened, but particularly in parts where small portions of calomel were discovered. These parts were very tender, and even the whole mucous coat could be separated with great ease. This organ contained about half a pint of dark-coloured fluid, like the sediment of port wine, and destitute of any remarkable odour. The spleen was four times larger than usual. This enlargement was not of recent origin; for the coat of the spleen was flaccid, thickened and of a white colour from ancient inflammation; so that there was reason to believe this organ had been much larger than at the time it was examined. Perhaps this derangement of the spleen was the cause of the dyspeptic symptoms. The liver was lighter coloured, and harder than usual, and exhibited a granulated appearance. The gall bladder was half full of bile, and contained two concretions of considerable size. The cystic duct was very much contracted, and showed the remains of three or four strictures, produced, no doubt, by the passage of gall stones, of which the sedentary habits of the patient allowed the formation. The omentum was full of fat. The kidnies swelled with fluid blood, and the urinary bladder contracted. The bladder was universally very dark coloured and fluid.

I am informed that two similar cases, examined about the same time, presented very similar appearances in the lungs.

No one will probably hesitate to call this disease a most violent inflammation of the pleura. From the common pleurisy, however, it differed in the extreme disproportion of the symptoms of inflammation, compared with those of the latter disease. This lowness of character will naturally lead us to arrange it under the head of the epidemic, which prevailed in the early part of the season among the soldiers of the United States army, and which has since extended to various parts of the country, an epidemic which seems allied, by some of its most peculiar symptoms, to the disease that that has been known under the name of petechial fever. The most remarkable circumstance in this case, is the very sudden appearance of the tumour on the neck, and its still more sudden disappearance. This phenomenon might seem incredible, were its existence not attested by two physicians of the highest respectability.—About the time this case occurred, and for some weeks previous, an uncommon epidemic had prevailed in this place. The principal symptoms were a swelling of the salivary glands, most commonly of the submaxillary; an inflammation of the mucous membrane of the tongue and mouth, with numerous small painful ulcerations; much fever, and sometimes a cutaneous eruption. The complaint continued about five days and subsided, whether medicine had been administered or not. It was, however, evidently mitigated by cathartics. In some instances, though rarely, the tumid glands suppurated. Although we cannot trace any chain of connection between this complaint and that of our case, the similarity in the glandular affection will perhaps justify the mention of it.—Physicians have often noticed the singular connexion between an epidemic disease and other disorders existing at the same time; and the influence which the former seems to have over the latter, in impregnating them, in a greater or less degree, with its peculiar character. Such a connexion we had an opportunity of witnessing in the year 1809, when the petechial disease proved so fatal in the country, and made its appearance in this vicinity, and even in the town. At that time there appeared a great variety of cutaneous affections of a new and anomalous character; and I could satisfactorily observe a chain of them extending from the malignant and frightful lividity of petechiæ, to the benign appearance of an ephemeral rash; while the other symptoms of disease, connected with these eruptions, were sometimes wholly different, and sometimes intermixed. Another circumstance, which was then noticed, is too remarkable to be omitted in this place. The petechial disease, in every severe instance, completely deranged the functions of the brain; and when that organ was examined after death, its membranes exhibited marks of inflammation; a quantity of serous fluid always appeared on the surface of the brain, and sometimes in the ventricles, in as great proportion as is observed in certain cases of hydrocephalus. At the very period this disease existed, and for some time after it had disappeared, the common hydrocephalus internus was observed, with its usual symptoms, in a far greater number of instances than had been known within the same space of time.—*New-England Journ. of Med. and Surg.* 1813.

